# Ordinary Optical Fiber Sensor for Ultra-High Temperature Measurement Based on Infrared Radiation

**DOI:** 10.3390/s18114071

**Published:** 2018-11-21

**Authors:** Qijing Lin, Na Zhao, Kun Yao, Zhuangde Jiang, Bian Tian, Peng Shi, Feng Chen

**Affiliations:** 1 Collaborative Innovation Center of High-End Manufacturing Equipment, Xi’an Jiaotong University, Xi’an 710054, China; xjjingmi@163.com (Q.L.); zdjiang@mail.xjtu.edu.cn (Z.J.); 2 State Key Laboratory of Mechanical Manufacturing Systems Engineering, Xi’an Jiaotong University, Xi’an 710049, China; vinsent@stu.xjtu.edu.cn (K.Y.); t.b12@mail.xjtu.edu.cn (B.T.); chenfeng@mail.xjtu.edu.cn (F.C.); 3 Electronic Materials Research Laboratory, Key Laboratory of the Ministry of Education & International Center for Dielectric Research, School of Electronic and Information Engineering, Xi’an Jiaotong University, Xi’an 710049, China; spxjy@mail.xjtu.edu.cn

**Keywords:** high temperature sensing, optical fiber sensor, infrared radiation

## Abstract

An ordinary optical fiber ultra-high temperature sensor based on infrared radiation with the advantages of simple structure and compact is presented. The sensing system consists of a detection fiber and a common transmission fiber. The detector fiber is formed by annealing a piece of ordinary fiber at high temperature twice, which changes the properties of the fiber and breaks the temperature limit of ordinary fiber. The transmission fiber is a bending insensitive optical fiber. A static calibration system was set up to determine the performance of the sensor and three heating experiments were carried out. The temperature response sensitivities were 0.010 dBm/K, 0.009 dBm/K and 0.010 dBm/K, respectively, which indicate that the sensor has good repeatability. The sensor can withstand a high temperature of 1823 K for 58 h with an error of less than 1%. The main reason why the developed ordinary optical fiber sensor can work steadily for a long time at high temperature is the formation of β-cristobalite, which is stable at high-temperature.

## 1. Introduction

Temperatures higher than 1000 °C represent an extremely harsh environment that can occur in various fields such as petrochemical engineering [1], metal and glass industries [2], and health monitoring of aero-engines [3]. The temperature will affect the stability of engines and mechanical structures, especially in high temperature environments. Accurate temperature information can provide basic guarantee for industrial production and condition monitoring of machines. However, high temperatures are often accompanied with other harsh conditions. For example, in oil production, the oil well environment not only involves high temperatures but also inflammable and explosive atmospheres [4]. The blades of large generators operate in high temperature and in a strong electromagnetic environment [5,6]. This puts forward specific requirements for high temperature sensors.

Optical fiber high temperature sensors with their advantages of high precision, good linearity, wide range of temperature responses, corrosion resistance, safety, anti-interference properties and so on can be applied to temperature measurement in severe environments involving severe vibration, strong electromagnetic interference, high temperature and pressure [7,8,9]. Optical fiber temperature sensors are mainly of two types: contact sensors and non-contact radiation sensors. The contact optical fiber temperature sensors mainly include Bragg grating (FBG) sensors [10], Fabry-Perot (F-P) sensors [11], Mach-Zehnder interference (MZI) [12] and Michelson sensors [13]. Although the sapphire fiber FBG sensor made by a femtosecond laser can work stably at 1500 °C [14] and can withstand 1900 °C in a short time [15], the contact optical fiber sensor can hardly work stably for a long time at high temperature due to the material and structure limitations. For example, FBG structures will be slowly eroded at high temperatures and lose their sensitive properties [16]. 

The radiant optical fiber temperature sensor has a high sensitivity at high temperature because the thermal radiation of objects increased exponentially with temperature. The high temperature sensors based on blackbody radiation theory are made of sapphire fiber that can withstand very high temperatures. Guo et al. [17] presented a sapphire fiber sensor made by processing metallic molybdenum film to develop the blackbody cavity. The measurement results of this sensor agreed with commercial temperature sensors in the range from 1760 °C to 1880 °C. Wangle et al. [18] presented a high temperature sensor fabricated by depositing a thin tantalum pentoxide film on the tip of a polished sapphire fiber. The sensor had a sensing range from 200 °C to 1000 °C. Li et al. [19] presented a sapphire fiber transient high temperature sensor based on blackbody radiation theory. The sensor could resist pressures over 50 MPa at transient temperatures of about 3000 °C. Shen et al. [20] presented a high temperature sensor made by doping Cr^3+^ in part of the fiber’s end and coating it with some radiant material. The sensing range of the sensor was up to 1800 °C. Zakharenko et al. [21] developed a new method of measurement to eliminate the influence of the blackness coefficient. Their pyrometers can be used to measure the temperatures of different melts in the range of 1100–2500 °C. However, forming a black body structures requires development of a temperature sensing medium ceramic layer [9,22], or sputtering precious metal temperature sensing media film [23,24]. This makes the development of the black body structures complicated and expensive. Moore et al. [25] developed a temperature sensor by applying a thin coating of a mixture of a high-temperature cement and nickel-oxide onto the tip of a silica optical fiber. This sensing tip became an isothermal cavity that emitted like a blackbody. The measurement range of the sensor was up to above 1000 °C. Levick et al. [26] presented a radiation thermometry method using photonic crystal fiber. The transmission of the sensor began to decrease at 934 °C. Thus, it is also difficult for blackbody thermometers which are made of silica optical fibers to measure high temperatures over 1000 °C. In this paper, an ordinary optical fiber high temperature sensor based on infrared radiation is presented. It is not only simple, compact, and convenient, but also can withstand high temperatures up to 1823 K for a long time. 

## 2. Design, Experiment and Discussion

**Figure 1:**
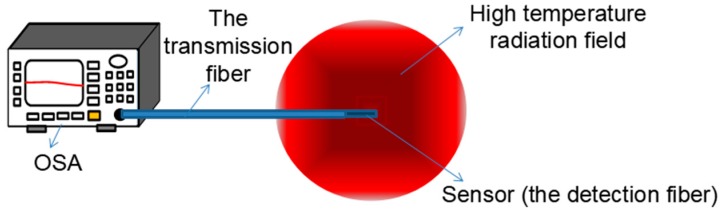
Schematic diagram of a optical fiber infrared radiation sensor.

The temperature measurement method is based on the principle of thermal radiation [17,18,19,20,21], that is, any object whose temperature is higher than absolute zero will emit infrared radiation to the outside. The infrared radiation energy of the object and the distribution of its wavelength are closely related to its surface temperature. Therefore, the surface temperature of the object can be accurately measured by measuring the infrared energy of its radiation. The schematic diagram of an optical fiber infrared radiation sensor is shown in Figure 1. A detection fiber, which is a crystallized fiber form by annealing twice at high temperature is fused to the end of transmission fiber. The thermal radiation energy of the high temperature field will be coupled to the end surface of the transmission fiber by a detection fiber and then transmitted to the spectral analyzer. According to the Planck’s radiation law [17,21,22], the change of temperature can be derived from the light intensity of the thermal radiation.

Multi-mode fiber (YOFC) with core diameter of 62.5 µm and cladding diameter of 125 µm was selected to fabricate the sensor. The fiber core is doped with Ge and F, and the cladding is pure quartz glass. One end of the fiber is wiped with alcohol and then a miller’s forceps is used to smooth it for use. The coating layer near the fiber cutting end is removed with miller’s forceps, then the fiber is wiped with alcohol absorbed on cotton to remove any residue and clean the end surface.

As the detection fiber a certain length of fiber is stripped off the coating layer using a cutter (FITEL S325, Furukawa, Shanghai, China). It is important to note that the fiber must be laid flat and the blade be perpendicular to the fiber axis during the cutting process. The detection fiber was fused to an ordinary single-mode fiber used as transmission fiber using an ordinary commercial fiber splicing machine (Furukawa S177B, Shanghai, China). The detection multi-mode optical fiber was placed in a high temperature furnace and went through two heating processes: heating to 1200 K for 8 min and cooling down to normal temperature, and then to 1500 K for 10 min and back to normal temperature.

**Figure 2:**
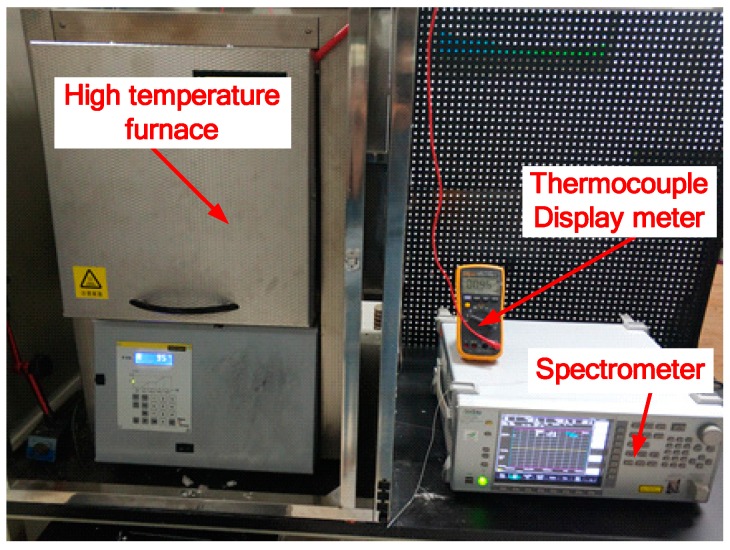
Static calibration experiment system of optical fiber infrared radiation sensor.

In order to determine the performance of the fabricated optical fiber infrared radiation sensor, a static calibration system was set up, as shown in Figure 2. The system includes a high temperature furnace with high temperature control precision (1 °C) whose highest temperature is 1600 °C and a spectrometer to record and analyze the temperature response of the sensor. The developed optical fiber infrared radiation sensor was put into the high temperature furnace for heating and the infrared radiation light detected by the sensor is transmitted to the spectrometer where the signal is collected and stored. The furnace was heated to 1823 K, then held at this temperature for 24 h, then cooled down to room temperature, then heated up again to 1823 K, then held at this temperature for 34 h, and then cooled down to room temperature.

## 3. Results and Discussion

**Figure 3:**
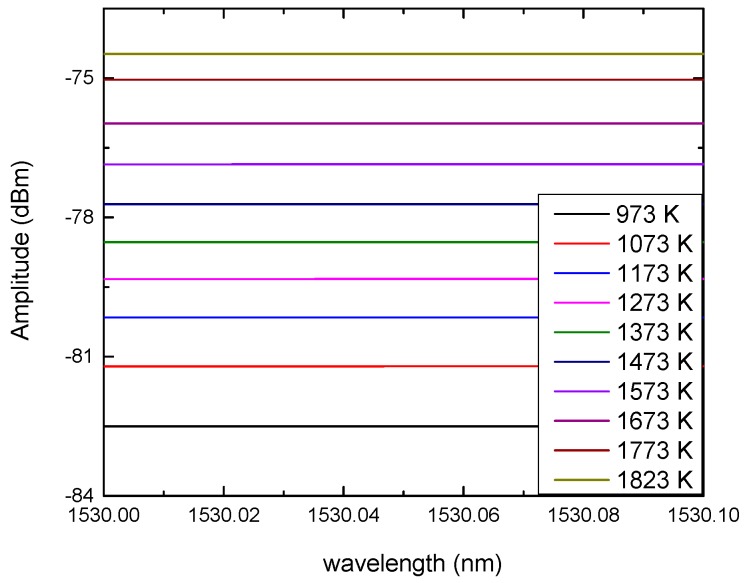
The output light intensity of the sensor collected by the spectrometer at different temperatures.

The output light intensity of the sensor collected by the spectrometer at different temperatures is shown in Figure 3. It can be observed that the intensity of infrared radiation increases as the temperature increases.

**Figure 4:**
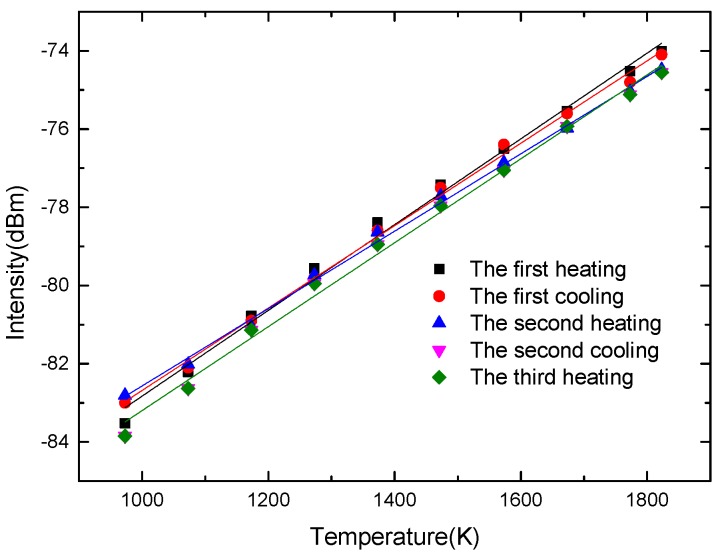
The temperature response curves of the optical fiber infrared radiation sensor in the three heating processes.

The temperature responses of the optical fiber infrared radiation sensor in the three heating processes are shown in Figure 4. The temperature response sensitivities of the same optical fiber sensor are 0.010 dBm/K, 0.010 dBm/K, 0.009 dBm/K, 0.010 dBm/K and 0.010 dBm/K, respectively. It can be obtained that the sensor has good repeatability.

**Figure 5:**
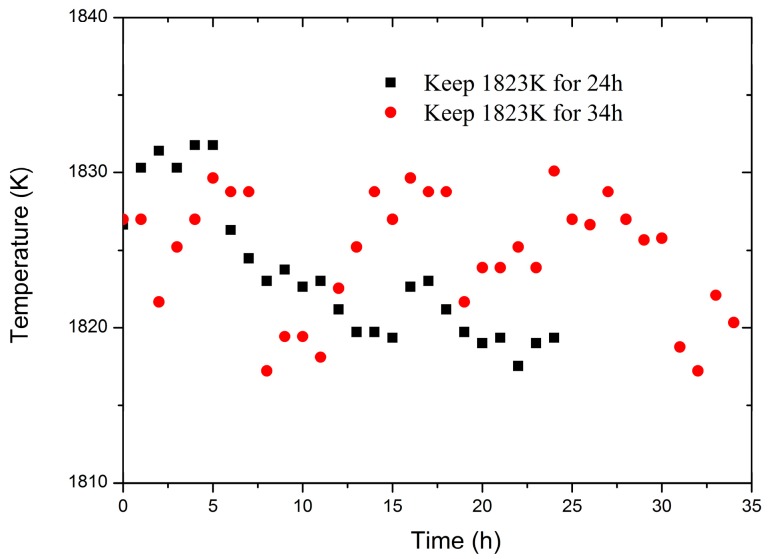
The temperature response of the optical fiber infrared radiation sensor keeping the temperature of 1823 K for 24 h and 34 h.

The temperature output responses of the sensor keeping the temperature at 1823 K for 24 h and 34 h are shown in Figure 5. The maximum temperature output value is 1831.4 K while the minimum is 1817.2 K, a deviation from 1823 K of 8.4 K and 5.8 K, respectively. Although the temperature output of the sensor fluctuates slightly near 1823 K, the temperature variation range is within 14.2 K, and the error is less than 1%. This indicates that the sensor can work steadily at high temperature for a long time.

Compared with the typical sapphire fiber black-body radiation high temperature sensor, the preparation process of the sensor we developed is simpler. There are two main fabrication methods for sapphire fiber high temperature sensors based on black body radiation. One is to coat one end of a sapphire fiber with a thin layer of temperature sensing medium ceramic having a high emissivity, and form a microfiber temperature sensing cavity by high temperature sintering [17,18]. This temperature sensing medium must meet a series of demanding requirements, such as high temperature resistance, good stability, and solid combination with sapphire fiber. The other is to sputter a precious metal temperature sensing media film at one end of sapphire fiber to form a small volume temperature sensing black body cavity as the thermal sensing head [19,20]. In order to prevent the metal from volatilization at high temperature, a layer of Al_2_O_3_ protective film is usually steamed on the outer wall of the cavity. 

According to the existing literature, for other types of common optical sensors such as F-P sensors, MZI sensors and Michelson sensors, the maximum operating temperature should not exceed 1200 °. Although the softening point of fused silica is around 1700 °, experiments [9] have shown that the silica fiber begins to undergo a phase transformation to generate microcrystals at about 1100 °C. This will induce an obvious loss in transmittance that may be caused by the devitrification of the core fiber of a fiber with cladding, scattering light out of the core of a fiber or stress-induced loss from the crystal growth on the surface [9]. This is also the main reason why most ordinary optical fiber sensors can’t work for a long time at temperatures above 1200 °C.

**Figure 6:**
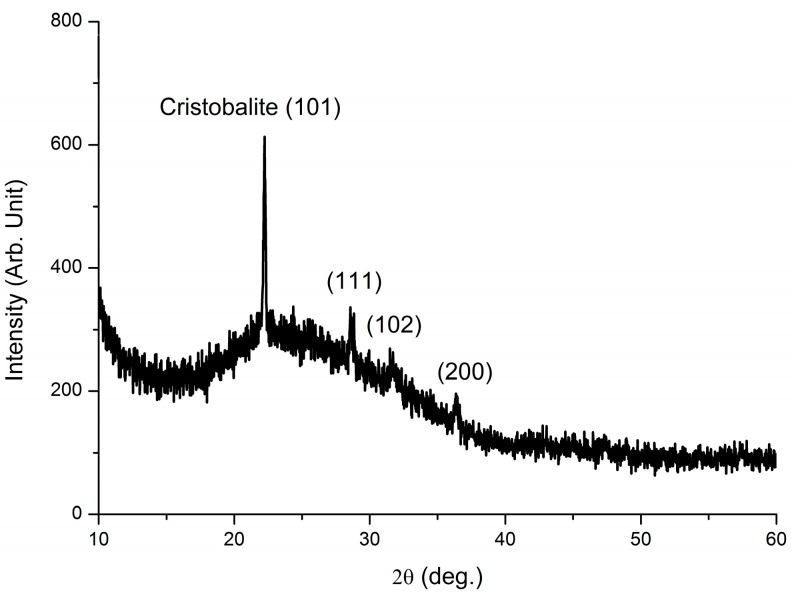
X-ray diffraction patterns of the crystallized optical fiber after two annealing treatments.

The crystallized optical fiber after two annealing treatments was ground into powder. X-Ray Diffraction (XRD) (Xpert Pro, Delft, The Netherlands) with a monochromatic Cu Kα (λ = 1.54 Å) source was used to analyze its crystallization structures. The XRD pattern is shown in Figure 6. It can be noted that the crystal structure of the optical fiber after two annealing treatments is significantly different from the ordinary optical fiber (silica fiber) which is amorphous. The crystal structure of the fiber after two annealing steps corresponds to α-cristobalite, which is the low-temperature tetragonal system phase of cristobalite [23,24,25]. The strongest diffraction peak occurs at 22.3° matching the first peak of cristobalite SiO_2_ crystal face (101) (PDF No. 82-1406). Some weak peaks are also observed at 28.8°, 31.9° and 36.5°, which are consistent with the (111), (102), (200), crystal faces of cristobalite respectively. It indicates that a new crystal form (cristobalite) has been created after annealing two times. 

**Figure 7:**
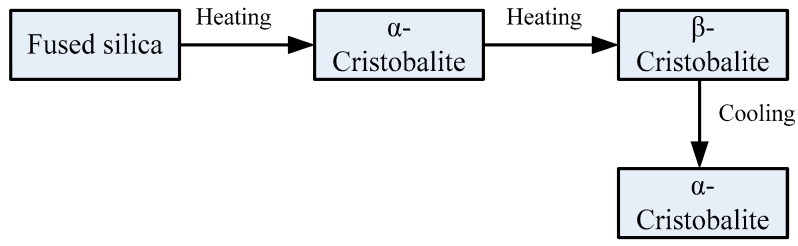
The crystal transformation in the fiber during the process of heating and cooling.

There are two varieties of cristobalite: high-temperature cristobalite with isoaxial crystal system (β-cristobalite) and a room temperature counterpart cristobalite (α-cristobalite) [23]. β-cristobalite stably exists at high-temperatures within the range of 1470–1728 °C and can transform to α-cristobalite at about 269 °C upon cooling down to room temperature. The crystal transformation in the fiber during the process of heating and cooling is shown in Figure 7. With the rising temperature, fused silica will transform into α-cristobalite which is the intermediate phase that cannot exist stably. As the heating temperature continues to rise, α-cristobalite will transform into β-cristobalite. Therefore, the β-cristobalite crystal structure of the optical fiber is the result of a high temperature process. This is the main reason why the developed ordinary optical fiber sensor can work steadily for a long time at high temperature. During the process of cooling to room temperature, β-cristobalite transforms into stabilized α-cristobalite, as shown in the XRD pattern (Figure 6).

The formation of cristobalite crystals is intrinsically affected by surface contamination and heating duration. It is worth noting that the formation of cristobalite crystals can be initiated by surface contamination [27,28]. In a practical situation such as the fabrication process, the detection fiber are often touched with the fingers and unclean tools, so it is probable that contaminants such as salt residue, cladding residue, powder left over from optical fiber cutting exist on the fiber surface and such contaminants become the origin of crystallization and considerably accelerate it [28].

**Figure 8:**
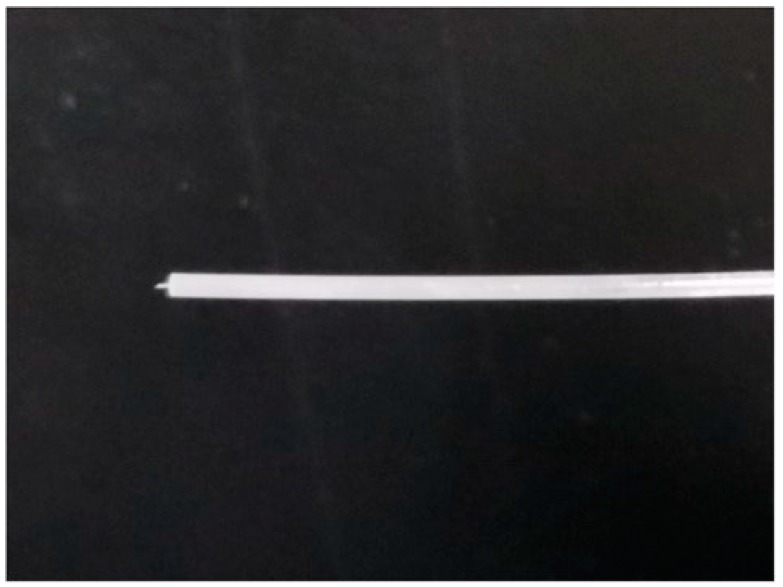
The crystalline fiber has been generated after annealing.

It has been experimentally determined that the growth rates at 1347 °C and 1540 °C for β-cristobalite in fused quartz are 6.5 × 10^−4^ μm/min and 4.1 × 10^−2^ μm/min [28], and the growth rate decreases as the annealing temperature is reduced. Supposing that the expansion of the crystal extends radially from the fiber surface to the center, for a fiber with the radius of 62.5 μm, the complete crystallization needed time is more than 96,153 min when the annealing temperature is below 1347 °C, which is far beyond the actual holding time at 1200 K and 1500 K. Therefore, fused silica, α-cristobalite and β-cristobalite coexist together in the two heat treatment processes. On the other hand, the time needed is less than 1200 min when the annealing temperature is above 1540 °C. Therefore, after 20 h at 1823 K the fiber is mainly in the form of β-cristobalite. In practice, this time may be shorter due to the promoting effect of surface contaminants. The sensor and fixed glass tube have changed from transparent to white crystals after annealing, as shown in Figure 8.

## 4. Conclusions

In summary, an ordinary optical fiber high temperature sensor based on infrared radiation consisting of a detection fiber and a transmission fiber was fabricated. The detection fiber was annealed twice at high temperatures of 1200 K and 1500 K, respectively. XRD results show that crystalline quartz was transformed into cristobalite including β-cristobalite that exists stably at high-temperature and α-cristobalite that exists at room temperature. Three heating experiments were carried out and the temperature response sensitivities are 0.010 dBm/K, 0.009 dBm/K and 0.010 dBm/K respectively. The sensor can withstand a high temperature of 1823 K for 58 h with an error of less than 1%. The main reason why the developed ordinary optical fiber sensor can work steadily for a long time at high temperature is the formation of β-cristobalite. The sensor has advantages of good performance, simple structure, compact, convenient and can be used to measure high temperatures in harsh environments.
